# Supplementary material for: Development of a computer-aided design software for the quantitative evaluation of aesthetic damage
Source: PLoS One. 2019 Dec 18;14(12):e0226322. doi: 10.1371/journal.pone.0226322 (PMC6919621; doi:10.1371/journal.pone.0226322)
Supplement: S1 Code site — 1. (ZIP) [file pone.0226322.s001.zip › [EESC_jr][Projeto]Relatorio_07_21/reports/templates_zip/gentelella-master/vendors/echarts/test/media-finance.html]

chart.setOption({backgroundColor: '#000'});

Then press restore buttton.

chart.setOption({backgroundColor: '#fff'});

chart.setOption({
series: [
{id: 'pie0', label: {normal: {position: 'inside'}}}
]
});

chart.setOption({
timeline: {
currentIndex: 5
}
});
